# Early CTA Diagnosis of Hemodynamically Occult External Iliac Artery Injury After Direct Anterior Total Hip Arthroplasty Treated with Covered Stent–Graft Repair: A Case Report

**DOI:** 10.3390/diagnostics16162488

**Published:** 2026-08-07

**Authors:** Maciej Błaszyk, Zuzanna Fryska, Jakub Waliszewski, Robert Juszkat

**Affiliations:** Department of Clinical Radiology, Poznan University of Medical Sciences, ul. Długa 1/2, 61-848 Poznań, Poland; zuzanna.fryska@usk.poznan.pl (Z.F.); rjuszkat@ump.edu.pl (R.J.)

**Keywords:** external iliac artery, vascular injury, total hip arthroplasty, computed tomography angiography, retroperitoneal hematoma, active extravasation, covered stent–graft, endovascular repair, Viabahn

## Abstract

**Introduction:** Major retroperitoneal arterial bleeding may remain clinically difficult to recognize when vital signs are initially stable. **Case presentation:** We report the case of a 67-year-old man who developed progressive abdominal and right groin pain radiating to the ipsilateral flank, accompanied by two episodes of vomiting and an early hemoglobin (Hb) and hematocrit decline on the first postoperative day after right-sided direct anterior total hip arthroplasty (THA). Despite the absence of hemodynamic instability, computed tomography angiography (CTA) demonstrated active contrast extravasation from the right external iliac artery (EIA) with a large retroperitoneal hematoma. CTA localized the bleeding source and enabled immediate endovascular treatment planning. Subsequent angiography confirmed active extravasation from the right EIA, and a covered stent–graft was deployed through ultrasound-guided superficial femoral artery access, achieving complete exclusion of the bleeding source with preserved arterial patency. The subsequent Hb nadir and transfusion were interpreted as reflecting preceding retroperitoneal blood loss rather than persistent bleeding, because follow-up CTA showed a stable hematoma without active extravasation. The patient was discharged in stable condition, and 1-month duplex ultrasound and following 3-month CTA confirmed stent–graft patency. **Discussion:** This case highlights the diagnostic mismatch that may occur between substantial retroperitoneal blood loss and initially reassuring hemodynamic findings. When progressive abdominal or groin symptoms are accompanied by a serial, otherwise unexplained Hb decline, CTA may be more informative than DUS for suspected deep pelvic bleeding because it can identify active extravasation, define hematoma extent, assess alternative abdominopelvic causes, and support treatment planning. In anatomically suitable lesions, covered stent–graft repair can rapidly control hemorrhage while preserving EIA patency. **Conclusions:** Stable vital signs do not exclude major retroperitoneal arterial bleeding after direct anterior THA. Progressive abdominal or groin pain with early postoperative Hb decline should raise suspicion for occult vascular injury, for which CTA can provide rapid diagnosis and guide immediate endovascular management.

## 1. Introduction

Vascular complications after total hip arthroplasty (THA) are uncommon but may be associated with substantial morbidity when diagnosis is delayed [1,2]. Early recognition can be particularly difficult in patients with retroperitoneal arterial bleeding because the initial symptoms may be nonspecific and vital signs may remain stable despite ongoing hemorrhage [1,3]. Several previously reported arterial injuries after anterior or direct anterior THA were recognized following overt limb ischemia, an abnormal postoperative vascular examination, or clinically evident deterioration [4,5,6,7,8]. A diagnostically challenging subgroup therefore comprises patients with preserved limb perfusion and stable hemodynamic parameters despite active arterial bleeding.

The direct anterior approach (DAA) provides an important anatomical context for this complication because anterior acetabular exposure is performed in proximity to the external iliac and common femoral vessels [4,6,9]. Injury may result from direct trauma, compression, or retractor-related mechanisms; however, the mechanism should be interpreted cautiously unless intraoperative vascular injury is directly observed [5,6,7,8].

In hemodynamically stable patients with suspected vascular injury, computed tomography angiography (CTA) can identify active extravasation, define the extent and location of retroperitoneal hemorrhage, localize the injured vessel, and provide anatomical information for endovascular treatment planning [3,10]. The clinically relevant feature of the present case was the discordance between major active external iliac artery (EIA) bleeding and the absence of shock, acute limb ischemia, or an abnormal distal vascular examination. We report a case of hemodynamically occult EIA injury after DAA THA in which early CTA established the diagnosis before clinical deterioration and enabled prompt covered stent–graft repair with preservation of arterial patency.

## 2. Case Presentation

### 2.1. Clinical Presentation and Diagnostic Trigger

A 67-year-old man with a history of chronic arterial hypertension underwent THA through a DAA using a bikini incision. During acetabular exposure, Hohmann retractors were positioned over the acetabular roof, at the superior and inferior borders of the femoral neck, and posterior to the greater trochanter. The circumflex vessels were identified and coagulated, and the acetabular preparation was performed after removal of femoral head and acetabular osteophytes. A cystic lesion of the acetabular roof was identified and reconstructed using autologous bone graft harvested from the femoral head before implantation of the acetabular component.

The operative report did not document excessive intraoperative bleeding, abnormal hemorrhage, hemodynamic instability, or any direct vascular injury. The exact estimated blood loss value was not available; however, no intraoperative event suggestive of vascular injury was reported. The immediate postoperative hemoglobin (Hb) and hematocrit (Hct) levels were 13.4 g/dL and 39.2%, respectively.

On the first postoperative day, the patient developed progressive abdominal pain radiating from the right groin to the ipsilateral flank, accompanied by two episodes of vomiting. At that time, he remained hemodynamically stable, with blood pressure of 113/67 mmHg and heart rate of 75 beats/min, without clinical signs of hemorrhagic shock. There were no clinical signs of acute limb ischemia.

Because the early clinical presentation was nonspecific, the initial differential diagnosis included gastrointestinal causes, such as food poisoning and appendicitis, as well as postoperative musculoskeletal pain, iliopsoas or retroperitoneal hematoma, bowel injury, and vascular injury. Leukocytosis and elevated C-reactive protein levels were initially considered compatible with either an inflammatory condition or a nonspecific postoperative response. However, these findings did not explain the progressive Hb and Hct decline.

Serial laboratory testing demonstrated a decrease in Hb and Hct to 11.6 g/dL and 33.5%, respectively. A repeat assessment approximately 3 h later showed a further decline to 10.6 g/dL and 30.7%. The combination of progressive abdominal and groin pain radiating to the ipsilateral flank and serial, otherwise unexplained Hb and Hct decline raised concern for concealed postoperative hemorrhage despite preserved blood pressure and the absence of clinical signs of shock and prompted urgent CTA on the same postoperative day.

DUS was not performed before CTA because the patient had preserved lower-extremity perfusion and no clinical signs of acute limb ischemia, whereas the principal diagnostic concern was a deep pelvic or retroperitoneal source of bleeding. In this setting, DUS would not have provided comprehensive evaluation of the retroperitoneal compartment or reliably assessed alternative intra-abdominal causes of the symptoms. CTA was therefore selected directly because it could simultaneously detect active bleeding, define the extent of any hematoma, identify the injured vessel, evaluate other abdominal and pelvic causes, and provide anatomical information for potential endovascular treatment planning.

### 2.2. CTA Findings

**Figure 1:**
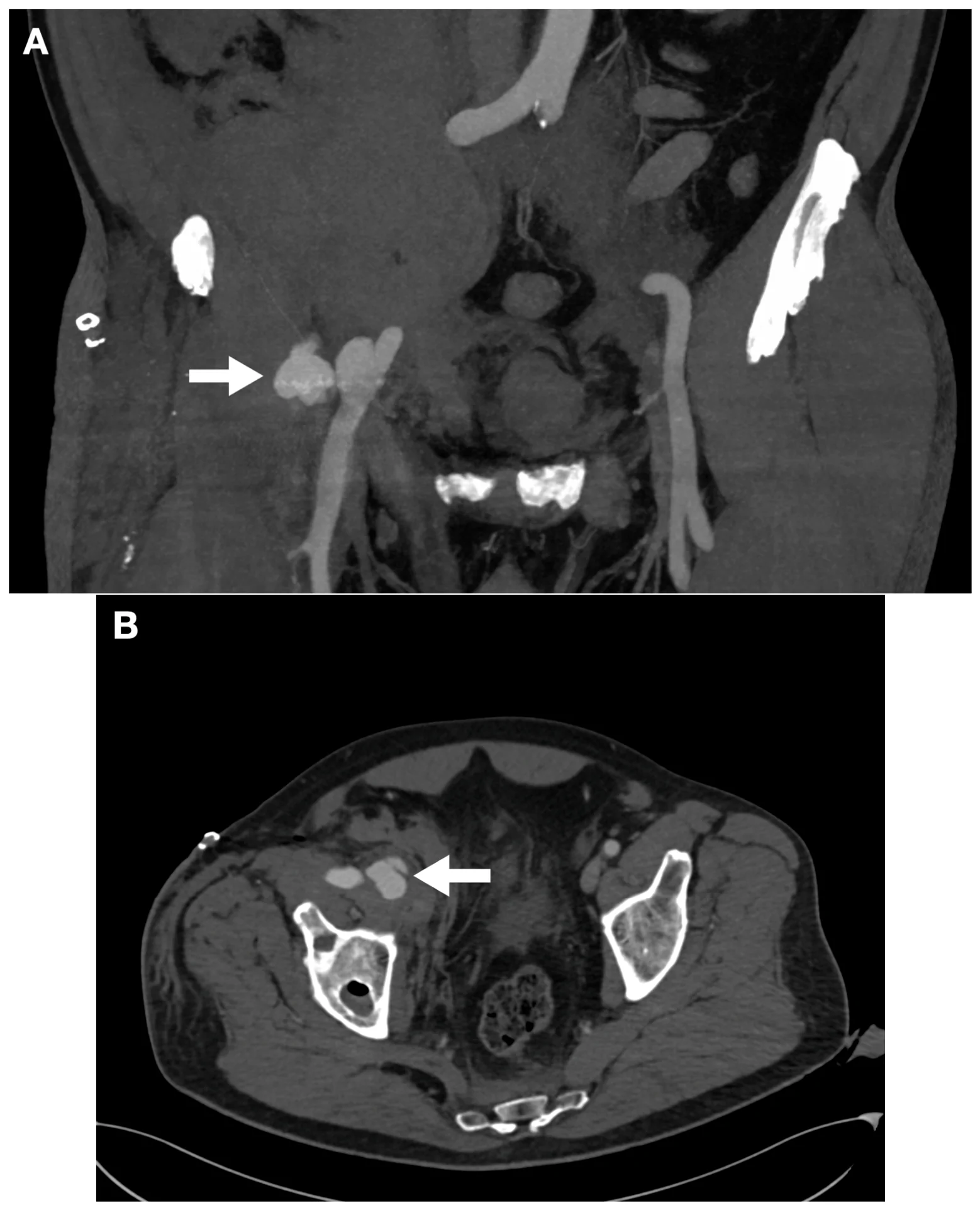

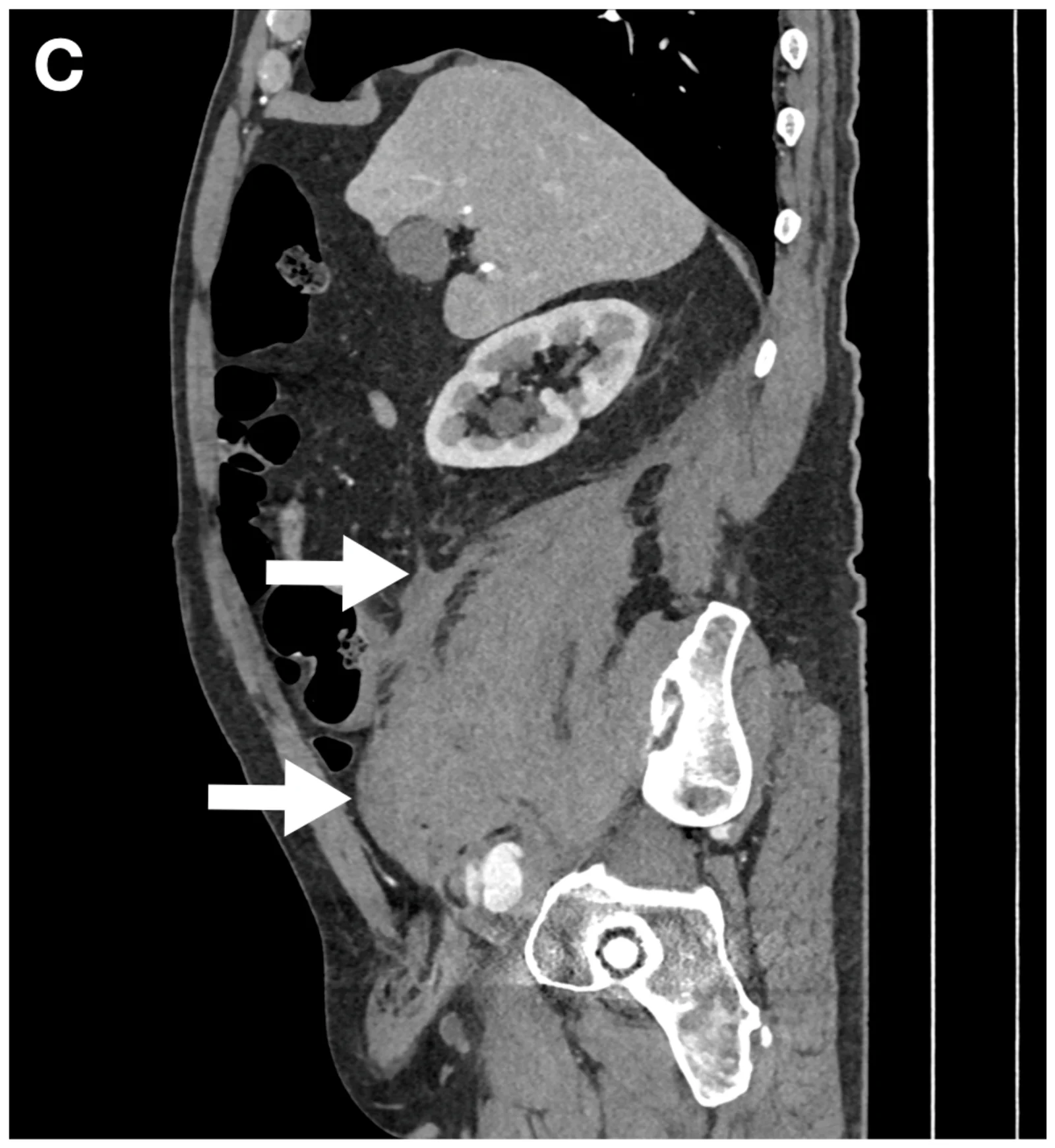
Computed tomography angiography (CTA) performed on the first postoperative day. (**A**) Axial arterial-phase CTA demonstrates active contrast extravasation arising from the right external iliac artery (arrow). (**B**) Coronal oblique/MIP reconstruction localizes the bleeding source along the right external iliac artery above the inguinal ligament at the level of inferior epigastric artery (arrow). (**C**) CTA shows the associated retroperitoneal hematoma extending along the right pelvic/iliac region (arrow). CTA enabled localization of the bleeding source and immediate endovascular treatment planning.

Computed tomography was performed using a multiphasic protocol comprising non-contrast, arterial-phase, and venous-phase acquisitions extending from the abdomen to the groins. In addition to evaluating the suspected source of concealed hemorrhage, the examination allowed assessment of alternative abdominal and pelvic causes of the patient’s symptoms. CTA demonstrated active contrast extravasation arising from the distal right EIA at the transition to the common femoral artery, in the region of the inguinal ligament, associated with a large retroperitoneal hematoma measuring approximately 12 cm × 8 cm × 30 cm (Figure 1). Active bleeding was identified by progressive contrast accumulation on post-contrast imaging. No additional source of active hemorrhage was detected.

CTA was decisive for diagnosis because it confirmed ongoing arterial bleeding, precisely localized the site of vascular injury, defined the extent of retroperitoneal hemorrhage, and provided anatomical information essential for immediate endovascular treatment planning. Based on these findings, the patient was referred for urgent angiographic confirmation and endovascular repair.

### 2.3. Endovascular Confirmation and Treatment

Immediately after CTA demonstrated active bleeding from the right EIA, the patient was referred for urgent angiographic confirmation and endovascular repair. Upon admission to the vascular surgery department, he remained without hypotension or clinical signs of shock, with a blood pressure of 137/82 mmHg and a heart rate of 103 beats/min.

Urgent endovascular treatment was performed. Under ultrasound guidance, vascular access was obtained through the right superficial femoral artery. The superficial femoral artery was selected to avoid manipulation within the potentially injured common femoral/external iliac segment and to provide a stable trajectory for precise stent–graft deployment. An 8F sheath was introduced. Systemic heparinization was withheld because of active bleeding.

**Figure 2:**
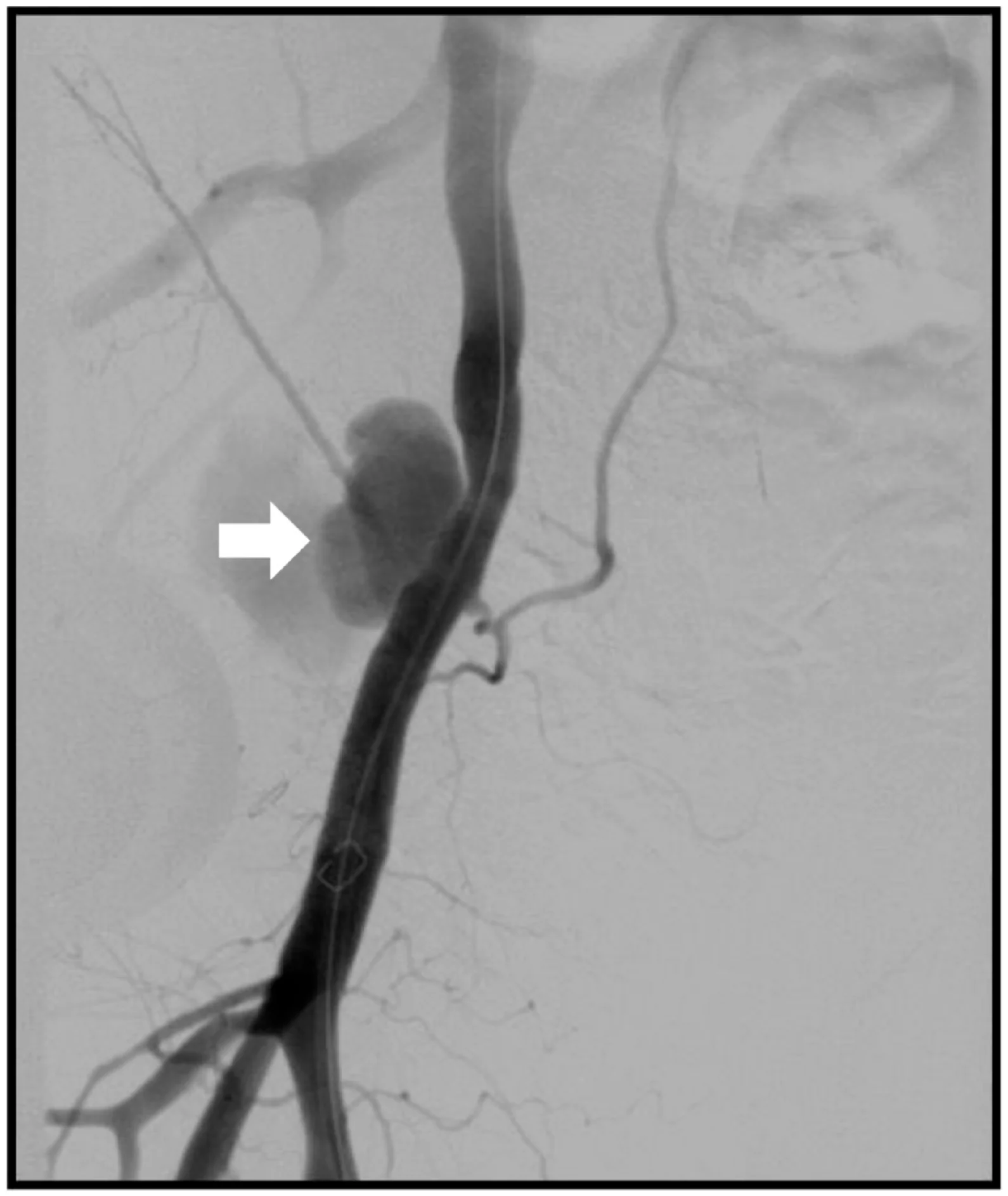
Digital subtraction angiography before stent–graft deployment confirms active extravasation from the right external iliac artery at the level of the inguinal ligament (arrow), corresponding to the bleeding site identified on computed tomography angiography (CTA).

**Figure 3:**
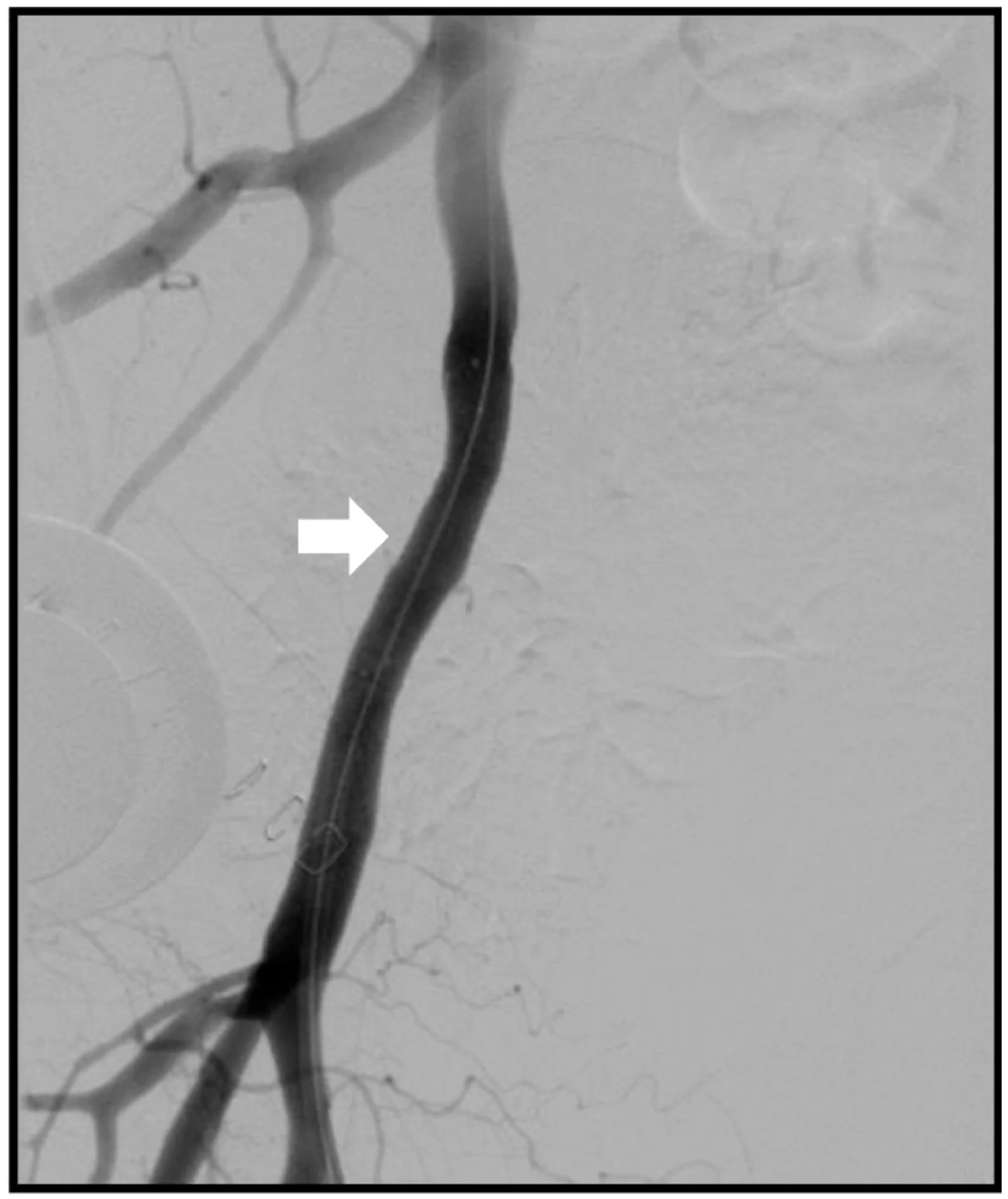
Completion angiography after deployment of a covered stent–graft (Gore Viabahn, 10 mm × 50 mm) across the injured segment of the right external iliac artery (EIA) demonstrates complete exclusion of the bleeding source, preserved external iliac artery patency, and no residual contrast extravasation (arrow).

Digital subtraction angiography (DSA) confirmed active extravasation from the right EIA at the level of the inguinal ligament, corresponding to the CTA findings (Figure 2). A covered stent–graft (Gore Viabahn, 10 mm × 50 mm, W. L. Gore & Associates, Inc., Flagstaff, AZ, USA) was deployed across the site of arterial injury. The stent–graft size was selected based on angiographic vessel diameter and available landing zones. Post-deployment angiography demonstrated complete exclusion of the bleeding source, preserved EIA patency, and no residual contrast extravasation (Figure 3). The vascular access site was closed using an AngioSeal closure device.

### 2.4. Postprocedural Course and Follow-Up

On the second postoperative day, corresponding to the day after successful endovascular exclusion of the bleeding source, Hb and Hct reached a nadir of 7.4 g/dL and 21.5%, respectively. The patient received two units of packed red blood cells. The delayed laboratory nadir was interpreted as reflecting the preceding retroperitoneal blood loss and subsequent equilibration rather than persistent arterial bleeding. This interpretation was supported by follow-up CTA, which demonstrated a stable retroperitoneal hematoma without active contrast extravasation.

The same follow-up CTA demonstrated a small access-site pseudoaneurysm measuring 10 mm × 11 mm. Because it remained clinically stable and showed no features requiring immediate intervention, it was managed conservatively.

Dual antiplatelet therapy (DAPT) with acetylsalicylic acid 75 mg and clopidogrel 75 mg was initiated 48 h after endovascular treatment after reassessment of bleeding risk, with the aim of maintaining stent–graft patency. Transition to single antiplatelet therapy was planned according to follow-up findings and bleeding risk.

On the sixth postoperative day Hb and Hct had increased to 11.8 g/dL and 34.7%, respectively. The patient was discharged in stable condition on the seventh postoperative day.

Duplex ultrasound (DUS) performed approximately 1 month after the procedure confirmed external iliac stent–graft patency, without evidence of right lower-extremity arterial occlusion or residual hematoma in the right groin region.

Moreover, approximately 3 months after the procedure, follow-up CTA also confirmed external iliac stent–graft patency and complete thrombosis of the previously identified pseudoaneurysm.

**Figure 4:**
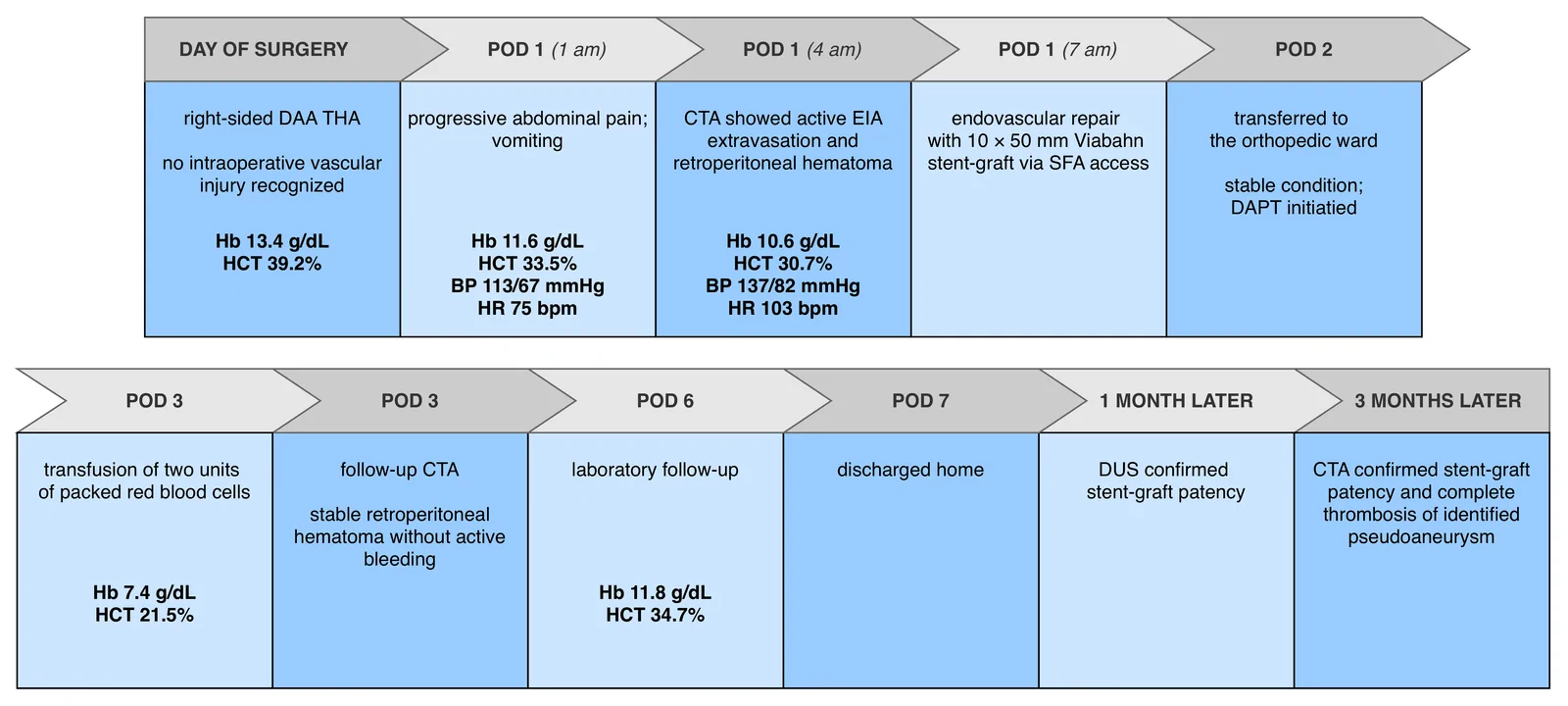
The clinical timeline illustrating the present case (Hb—hemoglobin, HCT—hematocrit, POD—postoperative day, CTA—computed tomography angiography, EIA—external iliac artery, SFA—superficial femoral artery, DAPT—dual antiplatelet therapy, DUS—duplex ultrasonography).

A clinical timeline illustrating the case is presented in Figure 4.

## 3. Discussion

The main diagnostic lesson of this case is that stable vital signs do not exclude major retroperitoneal arterial bleeding after direct anterior THA [1,2,3]. In the present patient, active bleeding from the EIA was not accompanied by overt hemodynamic instability or clinical signs of acute limb ischemia. The absence of hypotension or clinically relevant tachycardia in this patient illustrates why reliance on vital signs alone may delay diagnosis. Instead, the diagnostic trigger was the combination of progressive abdominal and groin pain, radiation to the ipsilateral flank, and an early postoperative Hb and Hct decline. This discordance between apparently stable clinical parameters and progressive symptoms should prompt consideration of occult vascular injury.

This presentation is clinically important because retroperitoneal bleeding may remain concealed until substantial blood loss has occurred [1,3]. In the early postoperative setting, abdominal or groin pain may initially be attributed to gastrointestinal, musculoskeletal, or nonspecific postoperative causes. However, when such symptoms are accompanied by an unexplained or progressive decline in Hb, vascular injury should remain in the differential diagnosis even in the absence of shock.

CTA was decisive in the present case because it demonstrated active contrast extravasation, confirmed the retroperitoneal location and extent of the hematoma, localized the bleeding source to the right EIA, and provided anatomical information for immediate endovascular treatment planning. It also enabled simultaneous assessment of alternative abdominal and pelvic causes of the patient’s symptoms.

DUS remains a rapid and valuable diagnostic tool when acute limb ischemia, an accessible pseudoaneurysm, or a localized femoral arterial lesion is suspected, and it is particularly useful for postprocedural surveillance. However, its diagnostic role depends on the suspected location and type of vascular injury. In a patient with preserved limb perfusion and suspected deep pelvic or retroperitoneal hemorrhage, DUS may not adequately evaluate the entire bleeding compartment, reliably determine whether bleeding is active, or comprehensively assess alternative intra-abdominal pathology. CTA was therefore selected directly as the most comprehensive initial imaging examination in the present clinical context [3,10,11].

Postoperative Hb decline is common after THA and should not, in isolation, mandate CTA. In our patient, the indication for CTA was based on the combination of progressive abdominal and groin pain, radiation to the ipsilateral flank, and a serial, unexplained decrease in Hb and Hct despite initially stable vital signs and preserved distal perfusion. This clinical pattern raised suspicion of concealed pelvic or retroperitoneal bleeding and justified further vascular evaluation. Therefore, CTA may be considered when progressive symptoms are accompanied by an otherwise unexplained postoperative Hb decline and occult vascular injury is suspected.

The exact mechanism of arterial injury in the present case remains uncertain. No intraoperative vascular injury was recognized, and the operative documentation did not report excessive bleeding, hemodynamic instability, or unexpected difficulties with hemostasis. Therefore, the absence of intraoperative warning signs highlights the potential for vascular complications after DAA THA to remain clinically occult during the index procedure.

Several technical aspects of the DAA may represent potential risk points because of the close anatomical relationship between the anterior acetabular region and the external iliac–common femoral vascular structures. Adequate acetabular exposure requires the use of retractors, and their position, depth, and direction of applied force may influence the displacement of adjacent soft tissues [12]. In the present case, Hohmann retractors were positioned over the acetabular roof and around the femoral neck to facilitate exposure. Although no direct vascular injury was observed, a retractor-related mechanism was considered anatomically plausible given the location of the arterial lesion adjacent to the anterior acetabular region.

Additional local anatomical factors may also have contributed to the complexity of acetabular preparation. In this patient, a cystic lesion of the acetabular roof required removal and reconstruction using autologous bone graft harvested from the femoral head. Although no intraoperative complication related to this procedure was documented, altered acetabular anatomy may potentially affect the relationship between the operative field, retractors, and surrounding vascular structures.

Nevertheless, the proposed mechanism remains hypothetical, as postoperative imaging cannot determine the exact intraoperative event responsible for arterial damage. The findings of this case should therefore be interpreted as emphasizing anatomical awareness and careful surgical technique rather than attributing the complication to a specific technical error.

The value of CTA in this case extended beyond diagnosis. Localization of the EIA injury allowed targeted angiographic confirmation and immediate endovascular repair. The use of superficial femoral artery access avoided manipulation within the potentially injured common femoral/external iliac segment and provided a stable trajectory for stent–graft deployment. A covered stent–graft enabled exclusion of the arterial defect while preserving EIA patency, which is essential due to its role as a major inflow vessel to the lower extremity [10,13,14,15,16].

Selected reports illustrating the heterogeneous clinical manifestations, diagnostic pathways, and management of arterial injury after THA are summarized in Table 1. The available cases include ischemic presentations diagnosed through postoperative vascular examination and DUS, overt hemorrhagic deterioration evaluated with CT, and mixed clinical series in which the detailed imaging pathway was not consistently reported.

Taken together, the selected reports demonstrate that arterial injuries after THA most commonly become apparent through overt limb ischemia, abnormal postoperative vascular examination, or clinically evident hemodynamic deterioration. In contrast, the present case illustrates that a major EIA injury may initially remain clinically occult despite preserved distal perfusion and stable vital signs. In this setting, progressive abdominal or groin pain accompanied by an unexplained serial postoperative decline in Hb should raise suspicion for concealed vascular injury and may justify urgent CTA.

The postprocedural Hb nadir and subsequent transfusion should be interpreted in the context of the preceding retroperitoneal blood loss rather than as evidence of persistent bleeding, because follow-up CTA showed a stable hematoma without active extravasation. This distinction is important when interpreting the early postoperative course after successful endovascular exclusion of the bleeding source.

Postprocedural antiplatelet therapy after covered stent–graft repair in this setting should be individualized [17]. In the present case, DAPT was initiated after reassessment of bleeding risk to support stent–graft patency. However, the optimal antiplatelet regimen after covered stent–graft treatment of iatrogenic iliac injury is not standardized and should be balanced against the risk of recurrent or ongoing bleeding.

This report has several limitations. First, it describes a single case, and the mechanism of injury was suspected but not intraoperatively confirmed. Second, the imaging follow-up is short, although 1-month DUS and 3-month CTA confirmed stent–graft patency. Longer clinical and imaging surveillance is planned to assess durable patency and exclude delayed stent-related complications.

## 4. Conclusions

Major EIA injury after direct anterior THA may remain hemodynamically occult despite active retroperitoneal arterial bleeding. In patients with unexplained progressive abdominal or groin pain accompanied by early postoperative Hb decline, stable vital signs should not exclude consideration of vascular imaging. Early CTA can detect active extravasation, define the extent of retroperitoneal hematoma, localize the injured artery, and guide immediate endovascular treatment planning. In anatomically suitable patients, angiographic confirmation followed by covered stent–graft repair may provide rapid exclusion of the bleeding source while preserving EIA patency.

## Figures and Tables

**Table 1:** Clinical presentation, diagnostic pathways, and management of selected arterial injuries after total hip arthroplasty, including direct anterior approach cases (CTA—computed tomography angiography; DAA—direct anterior approach; THA—total hip arthroplasty).

Study	Injured Vessel	Dominant Presentation	Hemodynamic Instability	Diagnostic Pathway	Treatment	Key Diagnostic Message
Mortazavi et al. (2019) [4]; three cases after primary DAA THA	Common femoral artery intimal injury with distal thrombosis	Immediate loss of dorsalis pedis pulse in two patients; delayed leg pain and pulse loss at approximately 10 h in one patient	No hemorrhagic instability or unusual intraoperative bleeding reported	Postoperative vascular examination → DUS demonstrating ipsilateral femoral artery occlusion; CTA and DSA not reported	Open exploration, Fogarty thrombectomy, and saphenous vein bypass in situ	Intimal CFA injury may present through immediate or delayed limb ischemia; repeated postoperative vascular examination and prompt DUS are important
Burlage et al. (2020) [5]; single case after DAA THA	External iliac artery	Intraoperative refractory hypotension and tachycardia requiring cardiopulmonary resuscitation	Yes—severe intraoperative hemodynamic collapse	Cardiac ultrasound → coronary angiography → laboratory confirmation of severe anemia → CTA demonstrating a large retroperitoneal hematoma and active distal EIA extravasation	Emergency open repair through retroperitoneal exposure and limited laparotomy with end-to-end EIA anastomosis	Major EIA bleeding may cause overt intraoperative shock and initially mimic a cardiac event; CT can identify the retroperitoneal source
An et al. (2018) [7]; single case after primary THA, approach not specified as DAA	Common femoral artery intimal injury with arterial thrombosis	Postoperative limb pain, numbness, decreased skin temperature, and loss of dorsalis pedis pulse	No hemorrhagic instability reported	Clinical vascular examination → emergency arterial ultrasonography demonstrating femoral artery occlusion → angiography confirming the lesion	Percutaneous recanalization and arterial stenting	Overt ischemic symptoms and pulse loss may direct the diagnostic pathway toward DUS followed by angiographic confirmation
Leiva et al. (2008) [8]; eight arterial injuries after primary or revision hip arthroplasty	External iliac artery (*n* = 3), common femoral artery (*n* = 3), internal iliac (*n* = 1), and deep femoral artery (*n* = 1)	Internal bleeding (*n* = 4), acute limb ischemia (*n* = 3), or subacute limb ischemia (*n* = 1)	Variable; patient-level hemodynamic data not reported	Detailed laboratory and imaging pathway not reported in the available report; vascular intervention occurred within <1 h to 20 days	Bypass, arteriography, primary repair, or thrombectomy with patch repair	Arterial injuries after hip arthroplasty have heterogeneous hemorrhagic and ischemic presentations; the diagnostic and treatment delay may vary substantially
Present case; primary DAA THA	Distal external iliac artery at the transition to the common femoral artery	Progressive abdominal and right groin pain radiating to the flank, vomiting, and serial Hb/Hct decline with preserved limb perfusion	No shock; initial BP 113/67 mmHg and HR 75 beats/min	Serial Hb/Hct decline → urgent CTA demonstrating active EIA extravasation and a 12 cm × 8 cm × 30 cm retroperitoneal hematoma → DSA confirmation	Covered stent–graft repair through ultrasound-guided SFA access	Major EIA bleeding may remain hemodynamically occult; CTA can establish the diagnosis and provide immediate endovascular treatment-planning information

## Data Availability

The data presented in this case report are available from the corresponding author upon reasonable request. Access to source clinical and imaging data is restricted due to patient privacy and institutional data protection regulations.

## Abbreviations

The following abbreviations are used in this manuscript:

CFA	Common femoral artery
CTA	Computed tomography angiography
DAA	Direct anterior approach
DAPT	Dual antiplatelet therapy
DSA	Digital subtraction angiography
DUS	Duplex ultrasound
EIA	External iliac artery
Hb	Hemoglobin
Hct	Hematocrit
THA	Total hip arthroplasty
